# Diabetes Mellitus among Adult Outpatients Visiting a Tertiary Care Centre: A Descriptive Cross-sectional Study

**DOI:** 10.31729/jnma.7027

**Published:** 2022-07-31

**Authors:** Bijay Khatri, Manish Kayastha, Rajan Shrestha, Janak Raj Bhattarai, Sangita Majhi, Sanjib Kumar Upadhyay, Madan Prasad Upadhyay, Kumud Kumar Kafle

**Affiliations:** 1 Academic and Research Department, B.P. Eye Foundation, Hospital for Children, Eye, ENT, and Rehabilitation Services, Madhyapur Thimi, Bhaktapur, Nepal; 2 B.P. Eye Foundation, Hospital for Children, Eye, ENT, and Rehabilitation Services, Madhyapur Thimi, Bhaktapur, Nepal; 3 KIST Medical College and Teaching Hospital, Gwarko, Lalitpur, Nepal

**Keywords:** *diabetes mellitus*, *hospital*, *Nepal*, *outpatient*

## Abstract

**Introduction:**

Diabetes mellitus is a chronic metabolic disease characterised by elevated blood sugar levels and is a pandemic of public health importance. Screening programs can help reduce morbidity and mortality by preventing or delaying complications. This study aimed to find out the prevalence of diabetes mellitus among adult outpatients visiting a tertiary care centre.

**Methods:**

This descriptive cross-sectional study was conducted among outpatients visiting a tertiary care centre between 1 January 2019 to 31 December 2019. Ethical approval was obtained from the Ethical Review Board (Registration number: 408/2020 P). Patients with unknown history of diabetes participating in free random blood sugar examinations were included in the study. Systematic random sampling was used. Point estimate and 95% Confidence Interval were calculated.

**Results:**

Among 385 adult outpatients, 17 (4.42%) (2.37-6.47, 95% Confidence Interval) had diabetes. The mean random blood sugar level of the diabetic patients was 281.41±57.49 mg/dl.

**Conclusions:**

The prevalence of diabetes mellitus among adult outpatients was similar to previous studies conducted in similar settings. Random blood sugar test in hospital outpatient settings is feasible to identify people with diabetes mellitus.

## INTRODUCTION

Diabetes is a pandemic of major public health importance, with an estimated global prevalence of 10.5%.^12^ The estimated prevalence of diabetes among 20-79 years old adult population in South Asia is 8.7%,^2^ whereas the estimated prevalence is 8.5% in Nepal.^3^ Mortality due to diabetes in Nepal is projected to increase from 8.68% in 2015 to 15.52% in 2040.^4^

Lack of proper care, poor awareness, and changing lifestyle will increase the future burden of diabetes in Nepal. Screening and earlier detection for undiagnosed cases are recommended to help prevent the disease from getting worse and minimise its impact on other health conditions, including mortality.^5^ Screening among hospital outpatients could be one of the methods in identifying missed cases of diabetes mellitus.

This study aimed to find out the prevalence of diabetes mellitus among adult outpatients visiting a tertiary care centre.

## METHODS

This descriptive cross-sectional study was conducted at Hospital for Children, Eye, ENT, and Rehabilitation Services (CHEERS), Bhaktapur, Nepal. The ethical approval was taken from the Ethical Review Board of Nepal Health Research Council (Registration number: 408/2020 P). The random blood sugar (RBS) level data from this screening program between 1 January 2019 to 31 December 2019 were reviewed. Patients aged 40-79 years who had no known history of diabetes were included in the study. Systematic random sampling method was used.

The sample size was estimated using the following formula:


$$\begin{array}{ll} \text{n} & ={\text{Z}}^{2}\times \frac{\text{p}\times \text{q}}{{\text{e}}^{\text{2}}}\\ & =1.96^{2}\times \frac{0.50\times 0.50}{0.05^{2}}\\ & =385 \end{array}$$

Where,

n = minimum required sample size
Z = 1.96 at 95% Confidence Interval (CI)
p = prevalence taken as 50% for maximum sample size calculation
q = 1-p
e = margin of error, 5%

The calculated sample size was 385. For systematic sampling, the sampling interval “k" was calculated using the following formula.


$$\begin{array}{ll} \text{k} & =\frac{\text{N}}{\text{n}}\\ & =\frac{6913}{385}\\ & =18 \end{array}$$

Where,

k = sampling interval
N= estimated total population during the study period
n = minimum required sample size

Using a computer-generated random number, '4' was selected as the starting point and then every 18^th^ item was selected till minimum sample size was reached.

Patients with RBS levels 200 mg/dl or higher were categorised as diabetic.^6^ The central obesity was assessed using waist circumference (WC), with WC ≥90 cm for males and WC ≥80 cm for females as overweight.^7^ The patients with blood pressure ≥130 and/or ≥80 mm of Hg were classified hypertensive,^8^ including those under medication for lowering blood pressure.

The records were imported into Microsoft Excel 2016. The data were checked for completeness, legibility, and consistency. The data were analysed using IBM SPSS Statistics 26.0. Point estimate and 95% CI were calculated.

## RESULTS

Among 385 adult outpatients, 17 (4.42%) (2.37-6.47, 95% CI) had diabetes mellitus. A total of 15 (88.24%) outpatients were in the age group 40-59 years old. The mean age was 51.65±8.26, 50.00±9.43, 52.80±7.64 years among all diabetic patients, females and males respectively. A total of 10 (58.82%) diabetic outpatients were males (Table 1).

**Table 1:** Age and sex-wise distribution of diabetic patients (n= 17).

Characteristics	n (%)
**Age group (years)**	
40-59	15 (88.24)
60-79	2 (11.76)
**Sex**	
Male	10 (58.82)
Female	7 (41.18)

The mean RBS level among diabetic patients was 281.41±57.49 mg/dl. The mean RBS level of diabetic male and female were 285.80±68.13 mg/dl and 275.14±42.12 mg/dl, respectively. The mean systolic blood pressure (SBP) and diastolic blood pressure (DBP) of diabetic patients were 122.97±12.18 and 80.00±5.00 mm of Hg, respectively. Similarly, the mean waist circumference of diabetic patients was 90.29±7.02 cm (Table 2).

**Table 2:** Clinical characteristics of diabetic patients (n= 17).

Characteristics	Male	Female	Total
	Mean±SD	Mean±SD	Mean±SD
SBP (mm of Hg)	124.00±12.65	121.43±14.64	122.97±12.18
DBP (mm of Hg)	80.00±4.71	80.00±5.77	80.00±5.00
WC (cm)	93.00±6.43	86.43±6.29	90.29±7.02

Among 17 diabetics, 12 (70.59%) were overweight and 6 (35.39%) were hypertensive (Table 3).

**Table 3:** Distribution of overweight and hypertension among diabetic patients (n= 17).

Characteristics	Male n (%)	Female n (%)	Total n (%)
Overweight	7 (41.17)	5 (21.41)	12 (70.59)
Hypertension	3 (17.64)	3 (17.64)	6 (35.39)

## DISCUSSION

In this study, the prevalence of diabetes mellitus among adult outpatients was 4.42%. This study analysed blood sugar level screening among outpatients with unknown diabetes status visiting for Eye or ENT consultation at CHEERS in 2019. CHEERS was selected purposively as this tertiary centre has regularly been conducting different non-communicable diseases screening programs for free at its health promotion unit since 2016.^9,10^

The prevalence in our study is similar to a study done during medical camp in Nepal, where 4.38% had similar blood sugar levels.^11^ A screening in Haryana, India, had 4% individuals with similar results.^12^ The differences in prevalence might be due to different age-groups and approaches of studies, though all were conducted in institutional settings. Our present study had selected patients using systematic random sampling method from age-group from 40-79 years coming for Eye or ENT consultation over one year, whereas the study in Haryana, India,^12^ was confined to a day's screening among all individuals aged 30 and above and study from Nepal,^11^ selected all 14-92 years old patients coming to the medical camp setting in a community hospital. All these studies had different age-groups of study participants, different methods of RBS investigation, and different lengths of the study. Despite these differences, all these studies show that screening for raised blood sugar levels is desirable for all patients regardless of their complaints in institutional settings.

In our study, males were more diabetic than females, which is similar to another study in a community hospital.^11^ Though RBS tests are not standard practice and fasting plasma glucose and postprandial plasma glucose are considered more definitive, such screening performed during outpatient visits is relatively inexpensive and convenient without requiring a prior fast.^13^ If this screening had not been done, these cases would have been left undetected or detected only after developing symptoms and/or complications of diabetes, given the paucity of symptoms in the early stages of diabetes.

The increasing burden of obesity has put the world on the brink of a catastrophic epidemic of diabetes.^14^ If a person is overweight, he/she should be considered for screening to assess the risk of hyperglycemia. A meta-analysis has concluded that people with elevated blood pressure are at increased risk of diabetes.^15^ This is evident from the fact that diabetes and hypertension share common metabolic pathways such as obesity, inflammation, oxidative stress, insulin resistance, and mental stress.^16^

This study is a result of screening of different non-communicable diseases at the hospital. The importance of the screening of hyperglycemia is highlighted by the fact that the undiagnosed cases are higher in developing countries,^17^ the asymptomatic phase may last for years,^5^ and can lead to macro-and micro-vascular complications, including neuropathy, nephropathy, retinopathy, coronary artery disease, stroke and peripheral vascular disease,^18-20^ and premature mortality,^21^ which can cause substantial economic loss for both the patient and added burden to the health system.^22^

The present study was conducted in a tertiary level Eye and ENT hospital. It is a single-centre study in an urban location and cannot be generalised to the larger population.

## CONCLUSIONS

The prevalence of diabetes mellitus among adult outpatients was similar to previous studies conducted in similar settings. The random blood sugar test for patients above 40 years could become a standard of practice in all health facilities with available laboratory services to timely diagnose this condition.
